# Interchangeability of external player load variables from different athlete tracking systems in English Premier League soccer players

**DOI:** 10.5114/biolsport.2026.153304

**Published:** 2025-08-05

**Authors:** Ronan Kavanagh, Kevin McDaid, Jack McDonnell, David Rhodes, David Tivey, Jill Alexander, Damian Harper, Piotr Zmijewski, Ryland Morgans

**Affiliations:** 1 Performance and Analytics Department, Parma Calcio 1913, 43121 Parma, Italy; 2 Football Performance Hub, Institute of Coaching and Performance, School of Health, Social Work and Sport, University of Central Lancashire, Preston, UK; 3 Applied Data Analytics Research Group, Dundalk Institute of Technology, Louth, Ireland; 4 Nottingham Forest FC, Nottingham, UK; 5 Jozef Pilsudski University of Physical Education in Warsaw, 00-809 Warsaw, Poland; 6 Research and Development Center Legia Lab, Legia Warszawa, Poland; 7 School of Sport and Health Sciences, Cardiff Metropolitan University, Cardiff, UK

**Keywords:** Exercise, Sports, Training, Football, Performance, Soccer

## Abstract

This study aimed to assess the interchangeability between tracking variables derived from Global Positioning System (GPS) and those of an Optical Tracking System (OTS) in elite soccer players. Twenty-six male professional outfield soccer players (age 27 ± 4 years, height 182 ± 6.67 cm, mass 80.73 ± 7.74 kg) from an English Premier League (EPL) team formed the sample. Positional information was recorded using a GPS (10 Hz Apex, STATSports, Belfast, UK) and concomitantly by an OTS using six semi-automated HD cameras sampling at a frequency of 25 Hz (Second Spectrum, Los Angeles, USA). While differences exist for both total distance and high-speed running (HSR) between the GPS and OTS, the GPS was highly correlated with the OTS (r^2^ > 0.99). The OTS displayed the highest values across all three examined variables. Total distance was 4% higher on average when utilising the OTS, while HSR and sprint distance were 12% and 18% higher respectively. Given its importance and the differences observed, regression equations should be utilised to align GPS and OTS data to allow practitioners to evaluate running performance and optimally prepare players for the demands of the game more effectively.

## INTRODUCTION

In soccer, training and match load monitoring is recognised as an important component of performance at the elite level [1]. External load in soccer matches has been studied in depth over the last two decades, which has improved knowledge on the demands of training and matches [2]. Many different technologies such as Global Positioning Systems (GPS) and Optical Tracking Systems (OTS) have been utilised to establish the physical profile of soccer players during matches with technological advancements allowing for a more detailed analysis of physical activity [3]. In terms of training and competition, GPS has been employed to measure, monitor and evaluate external load [4, 5]. Several aspects of players’ performance including speed and distances covered, in addition to the number of accelerations and decelerations during training sessions and matches have been analysed [6]. Moreover, OTS have been used to quantify the physical demands of players, while also including a multitude of different technical-tactical outcomes [7]. The information obtained from such systems supports practitioners in making decisions regarding training load prescription and manipulation [8]. Thus, optimising performance is a key underlying driver of the monitoring process [9].

FIFA now allows the use of GPS during competition, which provides data from a unified system and thus limits any interchangeability issues. Compliance, however, can be an issue with wearability in competitive matches and is practically often under-used [10]. Additionally, poor satellite signal can affect the validity and reliability of data recorded from the varying stadium roof heights and coverage [11]. Previously, elite soccer teams have utilised various league-wide OTS such as PROZONE® (Stats Perform), TRACAB® (ChyronHego), and Second Spectrum® (Second Spectrum Inc.). The evolution of tracking systems has forced practitioners to be critical when considering the validity and reliability of different metrics, particularly when combining data from multiple systems [8], such as GPS during training and camera systems during match-play. Previously Buchheit et al. [12] highlighted small differences (5.4%) between GPS and OTS concerning total distance covered, with OTS tending to report greater distance covered at higher speeds. Taberner et al. [13] further investigated the interchangeability of OTS across three competitive matches and found that PROZONE over-estimated sprint distance with a mean bias of 61% when compared to TRACAB. Similarly, Makar et al. [14] found that as velocity increases, the likelihood of encountering greater differences between the systems also amplifies.

Taberner et al. [10] further reports that data can be interchanged between GPS units and OTS without significantly influencing the interpretation of weekly load data. Furthermore, Ellens et al. [15] provided practitioners with equations to interchange variables between two different GPS and OTS. Currently, however, limited information exists regarding the interchangeability of data derived from GPS and OTS [13]. Understanding the relationship between systems is extremely important to accurately track player movement demands and support practitioners to improve performance and decrease injury risk. In addition, it is important to examine the interchangeability of data given the importance of monitoring training and match load [4, 16].

The categories most frequently employed to monitor training and match load are distances covered in specific thresholds [17]. In professional soccer, the management of high-speed running (HSR) (5.5–7 m/s) is of importance from a performance and injury prevention perspective [18]. Current literature has paid particular attention to these high velocity physical metrics to guide training approaches to optimise performance and reduce injury risk [19, 20]. While Bowen et al. [5] and Blanch et al. [21] both highlight the importance of monitoring HSR distance in injury prevention, research in Gaelic footballers by Malone et al. [22] suggested that players who were exposed to > 95% of individual peak velocity had a reduced injury risk when compared to players that were exposed to lower relative velocities.

Therefore, the purpose of this study was to assess the interchangeability of distance and speed tracking variables derived from GPS and an OTS in elite English Premier League (EPL) soccer players. The study hypothesis was that the application of regression equations will allow practitioners to successfully align the data, enabling practical interchangeability for load monitoring and decision-making purposes.

## MATERIALS AND METHODS

### Study Design

This observational study captured data over one season (2022/23) and included professional soccer players from one EPL team. Match running performance variables were collected using a GPS (Apex Pod, Statsports; Northern Ireland, UK) and OTS (Second Spectrum Inc, Los Angeles, USA). During the observation period, consistent player monitoring approaches were implemented without any interference from the researchers [23].

### Participants

Twenty-six male professional outfield soccer players (age 27 ± 4 years, height 182 ± 6.67 cm, mass 80.73 ± 7.74 kg) from an EPL team competing in the 2022/23 season participated in the study. The data was obtained from official matches played during the season (EPL n = 18). The research inclusion criteria have been previously applied [24] and were: (i) named in the first-team squad at the start of the study season, (ii) only completed official team training and matches during the study period. The sample group consisted of 26 outfield players (defenders n = 10, midfielders n = 10, and forwards n = 6). In the study season, 18 league matches were analysed, nine home matches and nine away league matches. A total of 212 individual match data points were examined with a median of 9 data points per player (range = 1 to 16).

Prior to data collection, participants were fully informed of the study design and provided informed consent. All data evolved from the players’ employment where routine monitoring over the course of the competitive season was conducted. Nevertheless, club approval for the study was obtained [25] and ethics was approved by the local Ethics Committee of University of Central Lancashire (BAHSS 646 dated 17/04/2019). The study followed the ethical guidelines for human study as suggested by the Declaration of Helsinki (2013). To ensure confidentiality, all data were anonymised prior to analysis.

### Data Collection

Tracking data was gathered simultaneously by a 10 Hz GPS (Apex Pod, version 4.03, 50 g, 88 × 33 mm; STATSports; Northern Ireland, UK) and an OTS (Second Spectrum Inc, Los Angeles, USA). Specifically designed vests were used to hold the GPS devices, located on the player’s upper torso, and anatomically adjusted to each player, as previously described [26]. All devices were activated 30-minutes before data collection to allow the acquisition of satellite signals and to synchronise the GPS clock with the satellite’s atomic clock [27]. Apex units have shown good levels of accuracy in sportspecific metrics in addition to non-significant and trivial differences when measuring peak velocity against the gold standard measure (Stalker ATS 2,34.7 GHz, United States) [28].

The GPS signal quality and horizontal dilution of position (HDOP) were connected to a mean number of 21 ± 3 satellites, range 18–23, while the HDOP was 0.9 ± 0.15, range 0.8–1.3. On completion of each match, GPS data were extracted using software (Sonra version 4.3.8, STATSports; Northern Ireland, UK) [29].

The installation process, reliability and validity of the OTS have been reported by FIFA electronic performance tracking systems programme [30]. Data was collected via semi-automated HD cameras with a sampling frequency of 25 Hz and positioned around the stadium. The OTS match data was processed directly using the Python programming language (Python 2.7) through the Spyder scientific development environment (https://www.spyder-ide.org/). Although match data can be imported and filtered through several commercially available systems including Sonra and OpenField (Catapult Innovations, Melbourne, Australia), processing the data directly via programmes such as Python 2.7 allows more accurate analysis [31]. For all matches, data was analysed for the full match duration including any stoppage time. Previously examined [23] and validated locomotive variables were included in the analysis; total distance (m), HSR distance (m; 5.5-7m/s) and sprint distance (m; > 7 m/s).

Weather data were collected for each EPL match analysed during this study. Across all fixtures, ambient temperature ranged from 18.0°C to 30.8°C, with relative humidity varying between 25% and 58%. Mean wind speed ranged from 9.0 to 16.7 km · hr^-1^, with gusts peaking at 29.6 km · hr^-1^. Barometric pressure during matches ranged from 1005.7 to 1026.5 hPa. All matches were conducted under standard professional conditions, and no adverse weather events were noted that could compromise GPS data quality or player performance.

### Data Analysis

Data were analysed through the RStudio statistical environment using appropriate libraries. The relationship between the GPS and OTS match values for total distance, HSR and sprint distance were examined using linear mixed models with the predictor match value treated as a fixed effect and the player and match treated as random effects. Predictor match values were scaled to allow for relatively large differences between players completing entire matches and non-starting players that had limited minutes as a substitute. Posthoc analysis was conducted considering the aims of the study.

### Statistical Analysis

A simple linear regression analysis was performed on datasets containing only independent measures to examine the relationship between variables across the two tracking systems. The derivation of an optimal model through the inclusion of slope and intercept parameters for fixed and random effects was explored using ANOVA with associated p-values and using Akaike Information Criterion (AIC). A p-value of 0.05 was set as the level of significance and in the derivation of confidence intervals (CI) for the fixed effect slope and intercept coefficients. The quality of the model was addressed using the r^2^ measure of standard error of the estimate (SEE). Correlation between the device measures was explored using repeated measures correlation values for both players and matches. The magnitude of correlations was defined by the following criteria: trivial (r ≤ 0.1), small (0.1 < r ≤ 0.3), moderate (0.3 < r ≤ 0.5), large (from 0.5 < r ≤ 0.7), very large (0.7 < r ≤ 0.9), and almost perfect (r > 0.9) [32]. To evaluate the existence of proportional bias, Bland-Altman methods were used, with the percentage difference between the devices regressed to the average of the device values [33].

## RESULTS

A descriptive analysis of the data is conducted across the four main variables and presented as the mean ± standard deviation.

Table 1 reports descriptive statistical match values for GPS and OTS. Note that paired t-test analysis indicated that all differences are significantly very strong, which is expected due to the large sample size.

**TABLE 1:** Mean ± standard deviation match values.

	Total Distance (m)	High-Speed Running (m)	Sprint Distance (m)	Peak Speed (m/s)
Optical Tracking System Mean ± SD	7366.69 ± 3426.94	434.52 ± 215.19	128.44 ± 85.97	8.46 ± 0.54
Global Positioning System Mean ± SD	7094.93 ± 3324.31	389.23 ± 194.65	108.81 ± 76.10	8.61 ± 0.59

Tables 2 and 3 show the linear regression analysis and regression equations for GPS to OTS and for OTS to GPS respectively for total distance, HSR and sprint distance. The details for the models with fixed effect intercept and slope estimate values (p < 0.0001), 95% CI for the slope, and the r2 value to measure the variation are explained by the model and the residual SEE for the model. As an illustration of the difference between the GPS and OTS measures, a total distance of 7094 m from the GPS would equate to 7415 m from the OTS, and an average sprint distance of 109 m from the GPS would equate to 131 m from the OTS.

**TABLE 2:** The model with fixed effect intercept and slope estimate values (p < 0.0001), 95% Confidence Interval (CI) for the slope, the r^2^ value to measure the variation explained by the model and the standard error of the estimate (SEE) for the model.

	Model	Slope	Intercept	95% CI	r^2^	SEE
Total Distance (m)	37.24 + 1.04 (GPS)	1.04	37.24	[1.03, 1.04]	0.99	56.9
High-Speed Running (m)	9.74 + 1.10 (GPS)	1.10	9.74	[1.08, 1.13]	0.98	21.1
Sprint Distance (m)	5.52 + 1.15 (GPS)	1.15	5.52	[1.11, 1.19]	0.98	9.7
Peak Speed (m/s)	1.34 + 0.83 (GPS)	0.82	1.36	[0.75, 0.90]	0.86	0.21

**TABLE 3:** Details for the model with fixed effect intercept and slope estimate values (p <0.0001), 95% Confidence Interval (CI) for the slope, the r^2^ value to measure the variation explained by the model and the residual standard error of the estimate (SEE) for the model.

	Model	Slope	Intercept	95% CI	r^2^	SEE
Total Distance (m)	0.96(OTS) – 32.8	0.96	-32.8	[0.96,0.97]	0.99	54.1
High-Speed Running (m)	0.89(OTS) – 3.44	0.89	-3.44	[0.88,0.92]	0.98	18.9
Sprint Distance (m)	0.85(OTS) – 2.72	0.85	-2.72	[0.82,0.88]	0.98	8.40
Peak Speed (m/s)	0.98(OTS) + 0.30	0.98	0.30	[0.91, 1.06]	0.86	0.23

**FIG. 1:**
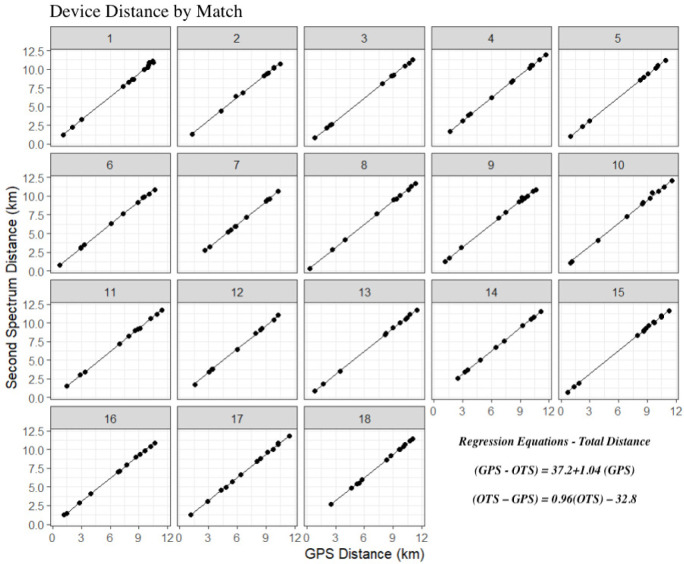
The relationship between total distance measures for each match including the regression line using the predictor measure and match identifier as covariates.

**FIG. 2:**
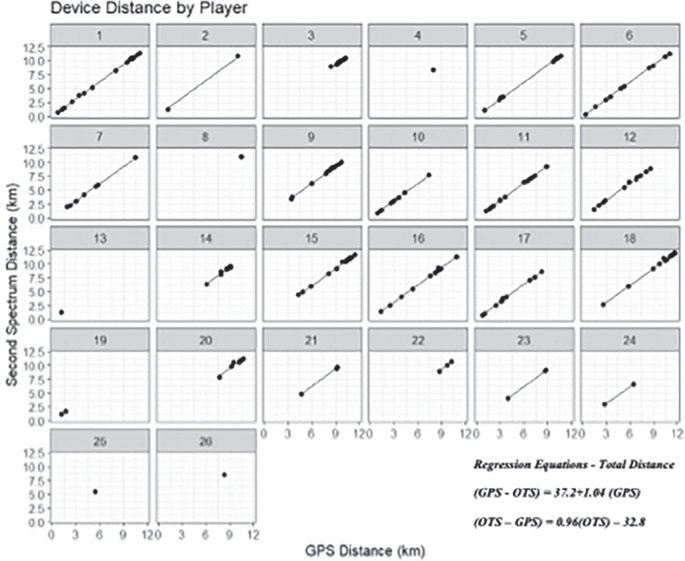
The relationship between total distance measures for each player including the regression line using the predictor measure and player identifier as covariates.

**FIG. 3:**
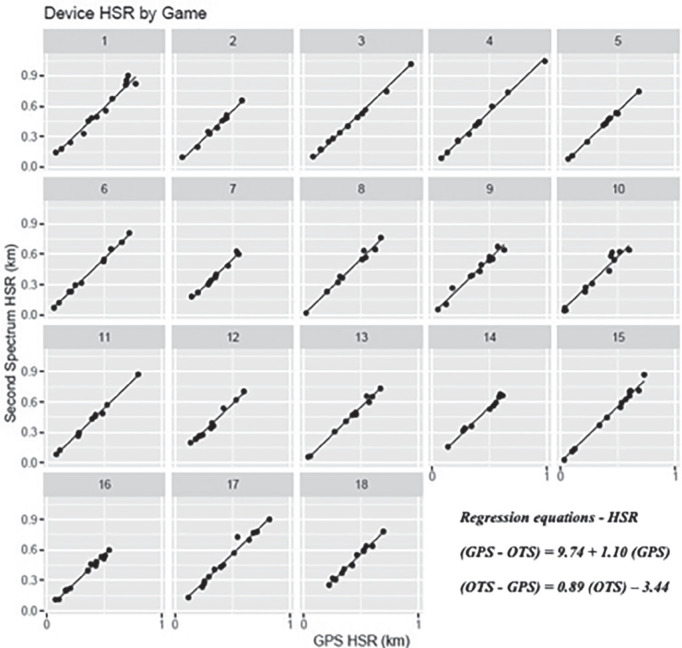
The relationship between HSR measures for each match including the regression line using the predictor measure and match identifier as covariates.

**FIG. 4:**
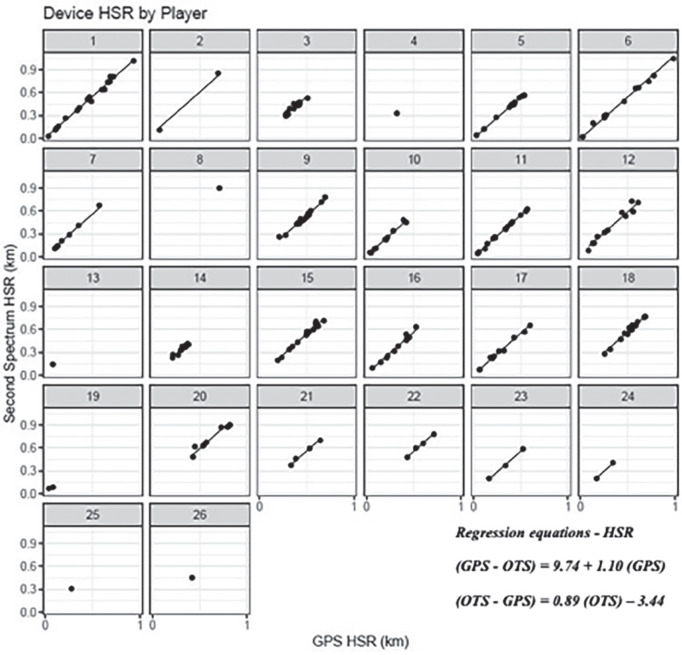
The relationship between HSR measures for each player including the regression line using the predictor measure and player identifier as covariates.

**FIG. 5:**
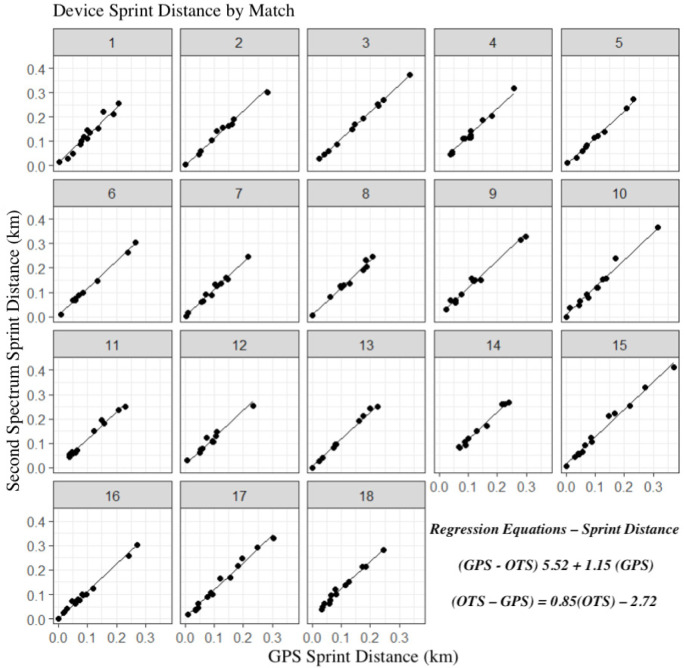
The relationship between sprint distance measures for each match including the regression line using the predictor measure and match identifier as covariates.

**FIG. 6:**
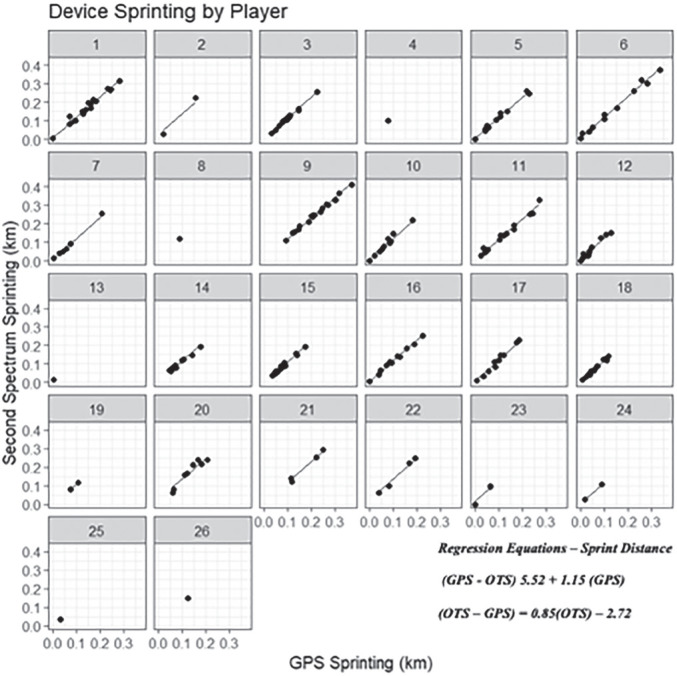
The relationship between sprint distance measures for each player including the regression line using the predictor measure and player identifier as covariates.

Figures 1, 3, and 5 display differences between measures for each match including the regression line using the predictor measure and match identifier as covariates. Figures 2, 4 and 6 illustrate the relationship between measures for each player including the regression line using the predictor measure and player identifier as covariates.

Additionally, to the results presented, Bland-Altman plots were generated and analysed. For total distance, the plots indicated a significant but minor proportional bias, with a slope of 0.000002 and a correlation of -0.33. For HSR, the slope of 0.00007 suggests a minor, marginally statistically significant bias, with a weak correlation of -0.15. Similarly, peak speed was marginally significant although a very low slope value of -0.008 and a weak correlation of -0.17 was observed. No significant proportional bias was observed for sprint distance. The magnitude of the bias slope coefficients suggests a very limited practical impact. The fact that some are significantly different from 0 is mainly due to the large sample size.

## DISCUSSION

The aim of the present study was to examine the interchangeability between tracking variables captured by a commercial GPS and data derived from an OTS. Results from the current study show that although the GPS was highly correlated with the OTS (r^2^ > 0.99), differences were observed between all tracking variables with the OTS displaying the highest values across all three variables. Total distance was 4% higher on average when using the OTS, while HSR and sprint distance were 12% and 18% higher respectively. The current findings are in agreement with Makar et al. [14] and Ellens et al. [15] who found that differences between systems increased as velocity increased. Despite this, peak speed was 2% lower when using the OTS as opposed to GPS.

These differences are most likely due to systematic error within the technology used to track positional variables [10]. For example, data filters such as a moving average can smooth the speed data causing a reduction in peak speeds [15] over the course of a full match, which can result in large differences between the two systems. Upper body sway when moving at high speed may affect OTS, while GPS units can shift in the vest when performing at high speed [15]. In addition, different stadia may have an impact on GPS data quality as satellite signal travels by line of sight and cannot pass through most solid objects such as high walls and curved stadium roofs seen in many modern stadia [34].

a key finding from this research is the differences observed in sprint distance between the two systems. The implications of these findings have a direct impact on training aimed at optimising performance and reducing the risk of inappropriate training load volumes in soccer players. The SEE in this study are smaller than those reported by Ellens et al. [15]. This discrepancy may be attributed to the larger sample size of the current study (18 EPL matches) compared to the five matches, comprising of two friendlies and three cup matches, examined by Ellens et al. [15]. It should also be noted that percentage error increases as speed increases as previously found [15]. This finding agrees with previous research, demonstrating that high-intensity measures are the least valid and reliable locomotive measure [13]. As a result, this study examined the raw data from both tracking systems. This notion aimed to remove some processing and filtering methods which may result in differences between systems.

The differences observed in sprint distance between the two systems are a key consideration for practitioners. For example, using the regression equation (Table 3), 400 m of sprint distance from OTS equates to 337 m in GPS data. Furthermore, during a week containing two matches, in the absence of regression equations, players could cover approximately 15% less GPS sprint distance than reported by the OTS. Discrepancies in such data may cause practical issues when planning training, as the common practice in professional soccer is to prepare players for the match demands. Despite the OTS displaying higher sprint distances when compared with the GPS, peak speed was higher when examining GPS data. Practically, this is vital information when considering the importance of nearpeak speed exposures in preparation for match demands and reducing injury risk [22, 35].

Knowledge of match running performance allows practitioners to optimise the training load according to the competition demands to improve players’ performance and reduce injury risk [36]. By accurately tracking sprint distances, fitness, medical and coaching staff can monitor player fatigue and manage workload more effectively. For example, a sudden increase in sprint distance over a short period (seven days) may indicate a higher risk of injury [20]. Accurate tracking data enables the implementation of load management strategies, such as reducing training load and intensity or providing additional recovery days to help mitigate injury risk. This proactive approach is essential for maintaining player health throughout a demanding season.

Understanding sprint distances may further contribute to strategic planning and tactical decision-making. By analysing tracking data, coaches can evaluate the effectiveness of tactical approaches and make necessary adjustments. For example, if a team’s defensive strategy involves high pressing, it is essential to know if players can sustain the required sprint intensity throughout the match. Indeed, the regression equations provided may be more important depending on the metrics examined. Given the importance of sprint exposure and the observed sprint distance differences, appropriate monitoring strategies and regression equations should be implemented with caution.

The present results have practical implications for coaches and performance staff. Analysis of weekly training loads within elite sports environments has now become commonplace [5]. However, unless this data correctly accounts for differences in tracking systems, practitioners may be exposing players to inappropriate training loads [13]. The key aspect to consider is the practical significance of the reported small differences in electronic performance tracking systems [10]. These subtle differences particularly at high intensities can significantly influence decision-making and data interpretation.

Regression formulae enable practitioners to align the data obtained from two different systems. Table 2 and 3 provide regression formulae as a practical tool for practitioners and researchers who may need to convert variables collected with GPS to OTS or vice versa. For example, if a practitioner converts HSR distance covered from the OTS to GPS the equation: GPS = 0.89 (OTS) – 3.44 may be applied. Thus, a player that covered 852 m of HSR in the OTS data may have only covered 755 m (0.89 (OTS) – 3.44 = 755 m) if the GPS system was employed. This difference of 97 m, accounts for a 12% difference and may have less practical significance when considering the volume of HSR distance covered. For example, 97m equates to a single pitch length run at high intensity and the velocity at which the distance was covered (5.5 and 7m/s). It is now commonplace for practitioners to monitor HSR distance over a microcycle [5] with players often covering up to 2000 m HSR [4]. Thus, a decrease of 97m in acute weekly load should not influence any training load-based decisions for sports science practitioners.

### Practical Applications

The regression equations outlined in this research provide practitioners with a means to align physical performance data from different systems. This is particularly useful for training prescription and the return to play process. The alignment of training and match data allows practitioners to prepare players for the match demands more effectively, reducing the risk of exposing the players to inappropriate training loads. Future studies should aim to identify the sources of error within different tracking systems. This could provide practitioners with more information surrounding the strengths and weaknesses of different systems and potentially inform the decision-making process. This future research may also help practitioners identify new technologies containing fewer sources of error.

### Limitations and Future Recommendations

Despite the strengths of this study, there are some limitations that should be acknowledged: a) the metrics chosen for this study did not account for accelerometery-based variables. The addition of acceleration and metabolic measures may provide practitioners with additional loading information, not provided by high-intensity distance metrics; b) data were collected in high-rise stadia environments, which may have attenuated GPS signal quality, a known challenge when recording locomotor data [11]. Hence, practitioners should interpret all data with caution in stadia and ensure raw traces of velocity and acceleration are analysed for irregularities generated by the GPS devices, which may include satellite signal loss leading to a delayed detection of locomotion [26]. Future research should examine HSR on a continuum as opposed to traditional speed thresholds. The binary classification of current speed thresholds can be affected by micro-differences between systems which can have a large influence on total HSR volume.

## CONCLUSIONS

The interchangeability between training and match load data is important to help practitioners effectively and confidently monitor and interpret the weekly volume of external running loads. This study demonstrates that running performance metrics, total distance, HSR, and sprint distance, measured via GPS are almost perfectly correlated with those obtained from a semi-automatic OTS (r^2^ > 0.99). Despite the strong correlations, systematic differences were observed, with the OTS consistently recording higher values that increased with running intensity. Specifically, the OTS recorded, on average, 4% greater total distance, 12% greater HSR, and 18% greater sprint distance compared to the GPS. The regression equations provided offer a practical tool for practitioners and researchers, enabling accurate conversion between data collected by an OTS and GPS.

Current findings demonstrate that match data can be interchanged between the present augmented GPS units and an OTS, in which the expected error would not have a practical influence on the interpretation of weekly load data when examining total distance covered and even HSR. Since the present commercial GPS and OTS are used universally within professional soccer clubs’, future research should focus on identifying and analysing the sources of error within each tracking system. This will enable practitioners to combine training (captured using GPS) and match activity (captured using OTS) data, to assist with the planning of appropriate training and recovery strategies to impact physical performance and potentially reduce injury risk.
